# Fluorine removal from sodium tungstate ion exchange effluent by precipitation with addition of lanthanum chloride

**DOI:** 10.3389/fchem.2023.1238644

**Published:** 2023-09-12

**Authors:** Linsheng Wan, Lifu Zhao, Caifang Cao, Dandan Gong, Xuepin Zeng, Liang Yang

**Affiliations:** Jiangxi University of Science and Technology, Ganzhou, Jiangxi, China

**Keywords:** fluorine removal, lanthanum chloride, precipitation, ion exchange effluent, sodium tungstate

## Abstract

The waste water generated from the sodium tungstate ion exchange process of scheelite hydrometallurgical extraction contains a certain concentration of fluorine ion, which caused environmental pollution and harmed human health. In this study, a new method for removing fluorine from the wastewater by precipitation with addition of lanthanum chloride was proposed. In the process, fluorine was removed by from the solution as insoluble lanthanum fluoride precipitates. To explore the favourable conditions for the formation of lanthanum fluoride, thermodynamic analysis of the La-F-H_2_O system was conducted. Results show that lanthanum fluoride is stable when the solution pH value is between 1.0 and 10.0, and the lanthanum fluoride is gradually converted into lanthana hydroxide when the pH value is more than 10.0 at 298K. The effects of various parameters on the fluorine removal were studied, and the optimum process parameters were determined. More than 92% of the fluorine can be removed when the concentration of fluorine in the solution ranged from 60 to 400 mg/L, the dosage of lanthanum chloride was 1.3 times of the theoretical amount, the pH value was 8.0 at 60°C for 30 min. After removing fluorine from the solution, the resiual fluorine concentrtion was lower than 10 mg/L, which could meet the requirement of national wastewater discharge.

## 1 Introduction

Scheelite is the main raw material of tungsten industry, which is usually associated with a certain amount of calcium fluoride (Dong et al., 2021; Wang et al., 2021; Liu et al., 2022; Zhang et al., 2022) At present, scheelite is almost treated by the process of “sodium hydroxide decomposition-anion exchange” to produce ammonium paratungstate (APT) (Zhao et al., 2013; Xue et al., 2019; Yin et al., 2020; Zhang et al., 2020). In the leaching process, scheelite was decomposed by sodium hydroxide to generate sodium tungstate solution, meanwhile calcium fluoride was also decomposed and entered the sodium tungstate solution as the form of sodium fluoride (Li et al., 2022; Yang et al., 2023). In addition, researchers had proposed to leach scheelite efficiently with the mixture of sodium phosphate and calcium fluoride, the obtained sodium tungstate solution also contained high concentration of fluorine (Gong et al., 2022; Yang et al., 2023). The sodium tungstate solution was then used to adsorbe tungstate ions by a strong base anion exchange resin (201 × 7 type) (Shen et al., 2018; Zhu et al., 2019). To obtain a high exchange capacity of tungstate on the resin,

$$2\bar{R_{4}\text{NCl}}+{\text{Na}}_{2}WO_{4}=\bar{{\left(R_{4}N\right)}_{2}{\text{WO}}_{4}}+2\text{NaCl}$$ (1)

the sodium tungstate solution needed to be diluted with about 10 times the volume of water in advance (Zhao et al., 2010). In the process of ion exchange, tungstate ions were adsorbed on the resin, while fluorine ions entered into the ion exchange effluent due to a weak binding ability of fluorine ion and the resin [Eq. (1)] (Cao et al., 2019). Since the sodium tungstate solution was diluted with water before ion exchange, the volume of ion exchange effluent discharged as wastewater was huge. About 100–120 m^3^ of wastewater was discharged for each ton of ammonium paratungstate production (Shen et al., 2019). The concentration of fluorine in the wastewater was approximately 100–200 mg/L (Liu et al., 2021). Discharge of the wastewater containing fluorine caused serious environmental pollution and endangers human health (Arahman et al., 2016; Zeng et al., 2019). According to the requirement of national wastewater discharge (GB8978-2002), the concentration of fluorine in the wastewater must be lower than 10 mg/L, so fluorine must be removed from the wastewater before discharging.

Compared with other kinds of wastewater containing fluorine, the sodium tungstate ion exchange effluent contains a large number of strong electrolytes (such as Na^+^and Cl^−^), making fluorine removal from the solution more difficult. At present, some methods for removing fluorine from the ion exchange effluent have been proposed, such as chemical precipitation, adsorption (Lv et al., 2021; Wang et al., 2021; Zhou et al., 2022; You et al., 2023). Due to the small solubility product constant of calcium fluoride (Ksp_CaF2_ = 2.7 × 10^−11^), calcium hydroxide was added into fluorine-containing wastewater to generate calcium fluoride precipitation for fluoride removal (Shi et al., 2015; Hu et al., 2021). However, the fluorine removal efficiency was not enough, and the residual fluorine concentration in wastewater was higher than 10 mg/L, which could not meet the national wastewater discharge standard. In addition, this method also had disadvantages of large amount of precipitated slag and slow defluorination speed. Based on the adsorption property of aluminum hydroxide to fluorine ion, activated aluminum hydroxide was used to remove fluorine from the wastewater (Wang, 2020; Li et al., 2021). However, owing to high concentration of chloride ion within the ion exchange effluent, chloride ions competed with the fluorine ions for adsorption, resulting in a low adsorption capacity of fluorine ions on the aluminum hydroxide. Therefore, the fluorine removal efficiency was not satisfactory. When the fluorine concentration was 82.5 mg/L in the wastewater, it was reduced to 38.2 mg/L after adsorption by activated aluminum hydroxide. In order to improve the fluorine removal efficiency, a two-step combined defluorination method was proposed to remove fluorine from the ion exchange effluent. First, calcium chloride solution was added to the ion exchange effluent to form calcium fluoride precipitates, and the fluorine concentration in the solution was reduced from 80 mg/L to 34.6 mg/L. Then, aluminum chloride was added into the solution for hydrolysis to generate active aluminum hydroxide to adsorb fluorine. The fluoride concentration was reduced to 8.3 mg/L by two-step combined defluoridation operation (He et al., 2020). Although the concentration of fluorine in the wastewater was lower than 10 mg/L, the process had the disadvantages of large chemical reagents consumption, large amount of defluorinated residue and long process flow.

Since the solubility product of calcium fluoride is not small enough, it is difficult to efficiently remove fluorine from the wastewater with calcium salt compounds. According to the chemical equilibrium theory, a solid of a lower solubility product facilitates the chemical precipitation reaction to proceed more efficiently. Therefore, we developed a new method for removing fluorine from the ion exchange effluent with addition of lanthanum chloride. The formation of LaF_3_ (Ksp_LaF3_ = 7 × 10^−17^) with a lower solubility product than that of CaF_2_ allowed the efficient removal of fluorine by one-step chemical precipitation. The chemical reaction of fluorine removal can be described as Eq. 2.

$${\text{LaCl}}_{3}+3F^{-}={\text{LaF}}_{3}↓+3{\text{Cl}}^{-}$$ (2)

The reaction equilibrium constant (K) at 298 K is calculated to be 1.05×10^31^, implying that fluorine ion can be theoretically completely converted into lanthanum fluoride precipitate. To remove fluorine from the ion exchange effluent efficiently, the thermodynamic analysis of fluorine removal was carried out, and the effects of process variables on fluorine removal were investigated in detail.

## 2 Experimental

### 2.1 Materials

The sodium tungstate ion exchange effluent in this study was provided by a tungsten plant in Ganzhou, Jiangxi province, and its main compositions are shown in Table 1. The chemical reagents such as lanthanum chloride, sodium fluoride, sodium hydroxide, hydrochloric acid used in this study were analytically pure.

**TABLE 1:** The main compositions of the ion exchange effluent.

Elements	Na	Cl	F	P	As	Si	pH
Concentration/mg/L	4,136	6,121	105.6	7.6	6.5	9.8	12.7

### 2.2 Experimental procedures

A certain volume of sodium tungstate ion exchange effluent was added into a conical flask, and then was placed in a thermostatic water bath pot with a stirring rate controller. The pH value of the solution was adjusted to a preset value with sodium hydroxide or hydrochloric acid solution. Sodium fluoride was used to adjust the concentration of fluorine in the solution. When the temperature of the ion exchange effluent in the conical flask reached a predetermined value, a certain amount of lanthanum chloride was added into the solution and stirred for a predetermined time. The slurry was removed from the conical flask and subjected to a filtration operation. The residue was washed with distilled water and dried at 80°C for 4 h. The volume and fluorine concentration of the filtrate were determined, and the ratio of fluorine removal was also calculated. The fluorine concentration in samples was determined by the selective ion electrode method.

## 3 Results and discussion

### 3.1 Thermodynamic analysis of fluorine removal by chemical precipitation with lanthanum chloride

In the process of fluorine removal from the sodium tungstate ion exchange effluent with addition of lanthanum chloride, solid reaction products such as LaF_3_, La(OH)_3_ can be thermodynamically formed. To provide theoretical guidance for fluorine removal, the thermodynamic analysis of the La-F-H_2_O system at 298 K was carried out. The possible chemical reactions in this system and the corresponding equilibrium constants are listed in Table 2 (Speight, 2005). The ionic concentration is used to instead of the ionic activity in the calculation due to the lack of the corresponding ionic activity coefficient. According to the law of mass conservation, the total amount of fluorine, lanthanum ([F]_T_ [La]_T_) satisfies the following equations:

$${\left[F\right]}_{T}=\left[F^{-}\right]+\left[\text{HF}\right]+\left[{\text{HF}}_{2}^{-}\right]+\left[{\text{LaF}}_{2}^{+}\right]+\left[{\text{LaF}}^{2+}\right]+\left[{\text{LaF}}_{3}\;\left(\text{aq}\right)\right]$$

$${\left[\text{La}\right]}_{T}=\left[{\text{La}}^{3+}\right]+\left[{\text{LaF}}_{2}^{+}\right]+\left[{\text{LaF}}^{2+}\right]+\left[{\text{LaF}}_{3}\left(\text{aq}\right)\right]+\left[\text{La}{\left(\text{OH}\right)}^{2+}\right]+\left[\text{La}{\left(\text{OH}\right)}_{2}^{+}\right]+\left[\text{La}{\left(\text{OH}\right)}_{3}\left(\text{aq}\right)\right]+\left[\text{La}{\left(\text{OH}\right)}_{4}^{-}\right]$$

**TABLE 2:** Thermodynamic equilibrium equations and constants of La-F-H_2_O system at 25°C.

No.	Equilibrium reactions	Constant K
4	${\text{LaOH}}^{+}$ = La^3+^+ ${\text{OH}}^{-}$	10^–5.34^
5	${\text{La}\left(\text{OH}\right)}_{2}^{+}$ = La^3+^+ ${2\text{OH}}^{-}$	10^–9.86^
6	$\text{La}{\left(\text{OH}\right)}_{3\left(\text{aq}\right)}$ = La^3+^+ ${3\text{OH}}^{-}$	10^–14.09^
7	${\text{La}\left(\text{OH}\right)}_{4}^{-}$ = La^3+^+ ${4\text{OH}}^{-}$	10^–15.14^
8	$\text{La}{\left(\text{OH}\right)}_{3\left(s\right)}$ = La^3+^+ ${3\text{OH}}^{-}$	10^–18.7^
9	HF = H^+^+ $F^{-}$	10^–3.17^
10	${\text{LaF}}^{2+}$ = La^3+^+ $F^{-}$	10^–3.85^
11	${\text{LaF}}_{2}^{+}$ = La^3+^+ ${2F}^{-}$	10^–6.65^
12	${\text{LaF}}_{3\left(\text{aq}\right)}$ = La^3+^+ ${3F}^{-}$	10^–8.69^
13	${\text{LaF}}_{4}^{-}$ = La^3+^+ ${4F}^{-}$	10^–16.2^
14	${\text{LaF}}_{3\left(s\right)}$ = La^3+^+ ${3F}^{-}$	10^–16.2^
15	$H_{2}O$ = H^+^+ ${\text{OH}}^{-}$	10^–13.99^

**FIGURE 1:**
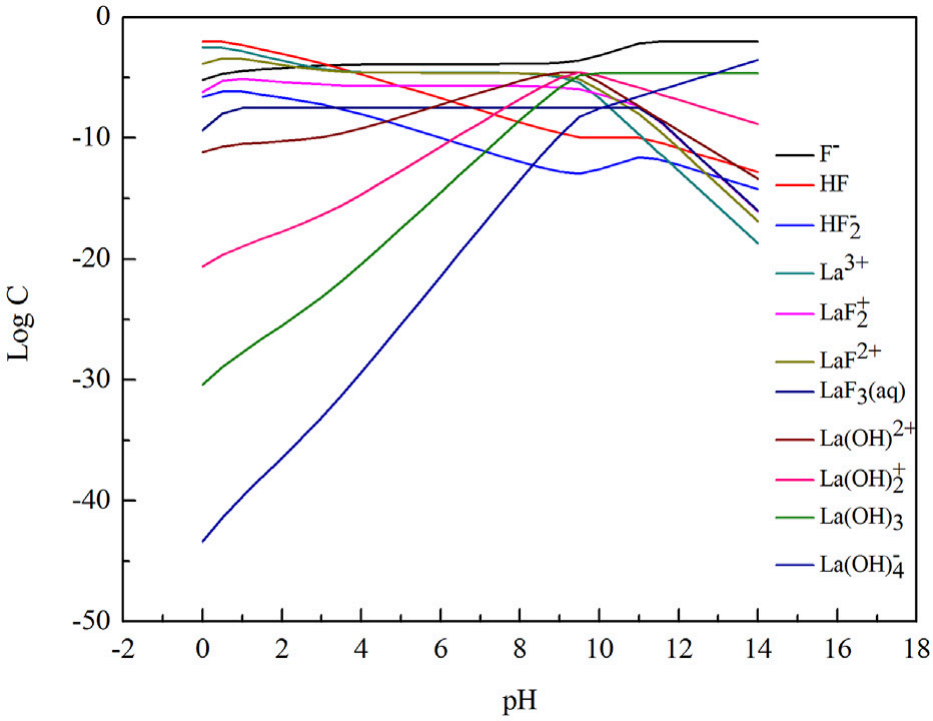
LogC-pH diagram of different ions in La-F-H_2_O system ([F]_T_ = 0.01 mol/L [P]_T_ = 0.0033 mol/L.

The form of different kind of lanthanum ion and fluorine ion is closely related to the pH value of the solution, so the concentration of different ions was calculated as a function of pH value, and the corresponding lgC-pH diagrams were plotted, shown as Figure 1. The calculation process was carried out by using the Newton iteration method.

In the La-F-H_2_O system, the order of the equilibrium solid phase is LaF_3_ and La(OH)_3_ with increasing pH value. When the pH value ranges from 1.0 to 10.0 (between line ① and line ②), the stable solid precipitate is LaF_3_. When the pH value ranges from 10.0 to 11.0 (between line ② and line ③), the stable solid precipitates are LaF_3_ and La(OH)_3_. When the pH value is over 11, La(OH)_3_ is formed as stable solid precipitate. LaF_3_ is stable over a wide pH range, implying that fluorine is easily removed by chemical precipitation with addtion of lanthanum chloride from a theoretical perspective.

**FIGURE 2:**
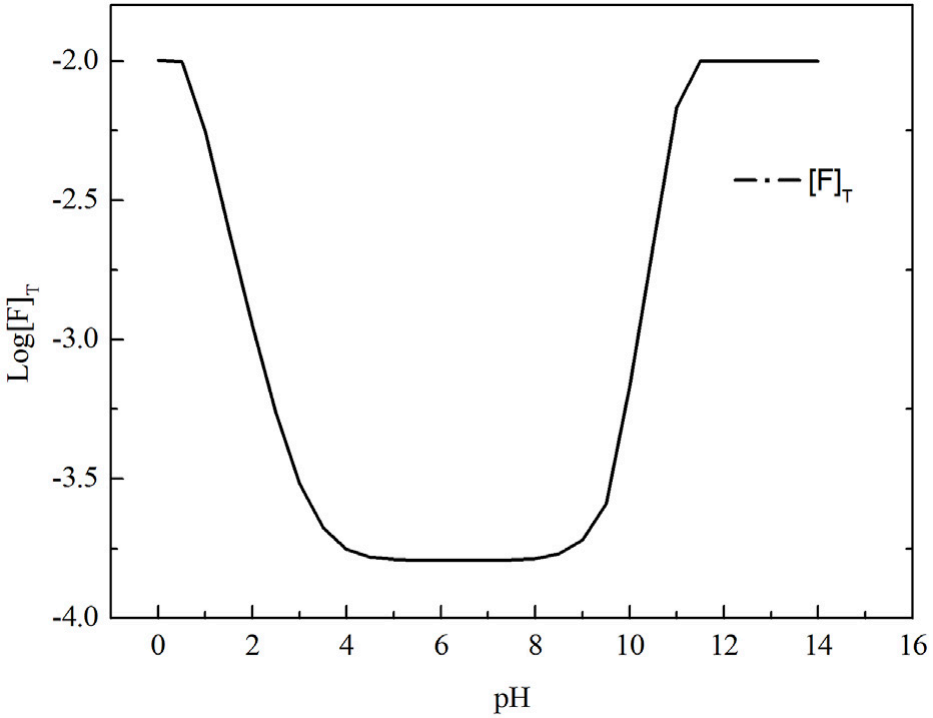
Variation of total fluorine concentration in the solution with pH value.

**FIGURE 3:**
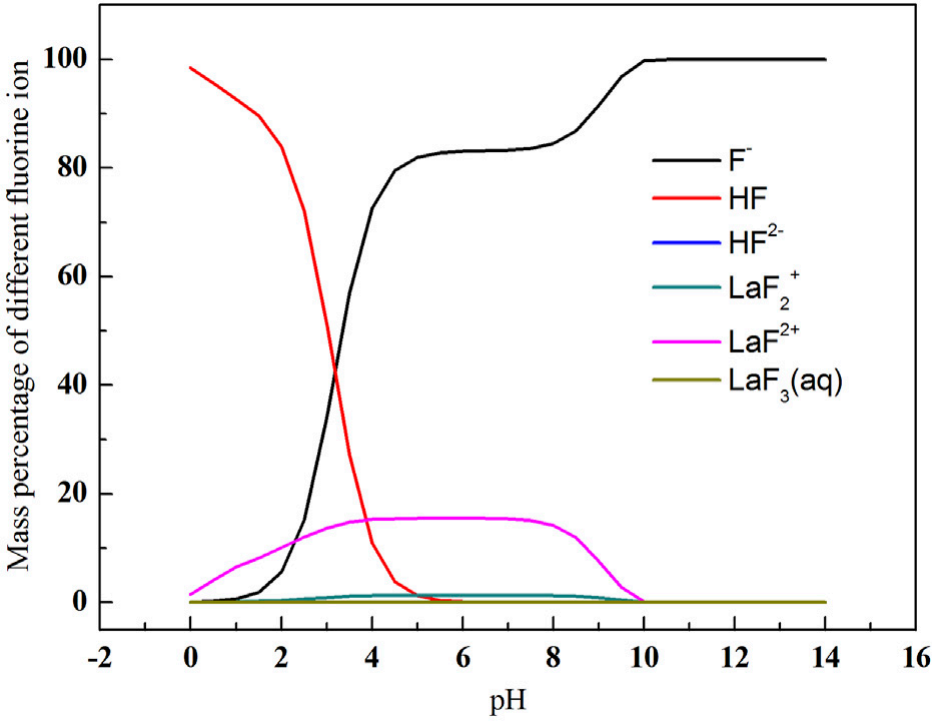
Variation of mass percentage of different fluoride-containing ions with pH value.

As shown in Figure 2, with the increase of pH value, total fluorine concentration in the solution first decreased and then increased. The total fluorine concentration in the solution was the lowest when the pH value ranged from 4.3 to 8.2, indicating that fluorine canbe efficiently removed in this pH range. As shown in Figure 3, when the pH value between 4.3 and 8.2, the mass ratio of F^−^, HF and LaF^2+^ is dominant. When the pH value is over 10.0, the total fluorine concentration increased sharply. Because the lanthanum fluoride is unstable and converts into lanthanum hydroxide, thus the total fluorine concentration rapidly increases. When the pH value is below 1.0, the lanthanum fluoride is converted into HF, so the total fluorine concentration also increases.

**FIGURE 4:**
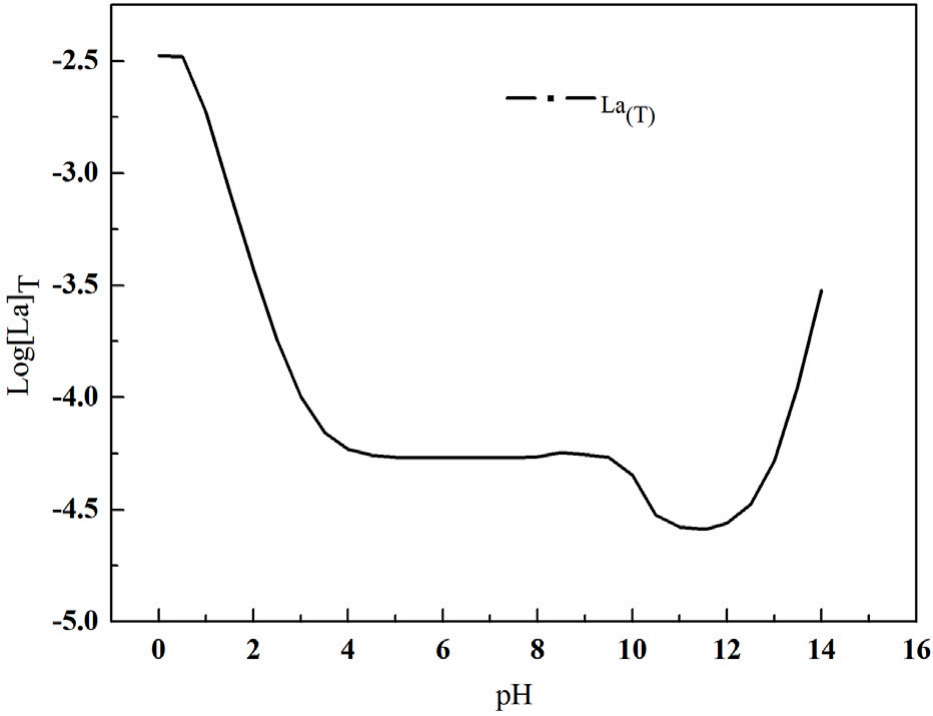
Variation of total lanthanum concentration in the solution with pH value.

**FIGURE 5:**
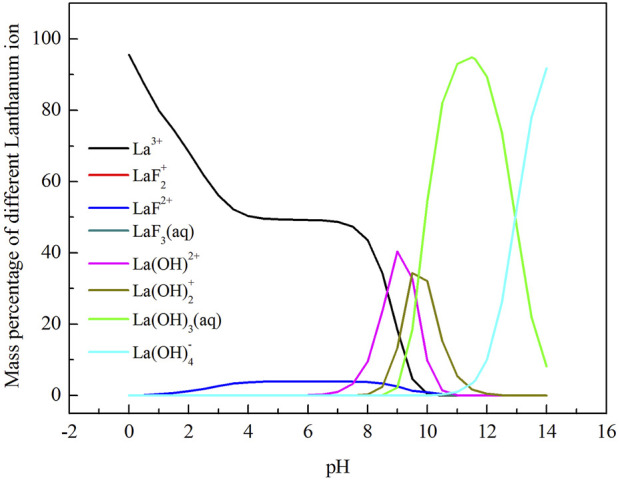
Variation of mass percentage of different lanthanum-containing ions with pH value.

As shown in Figure 4, with the increase of pH value, the total lanthanum concentration first decreased and then increased. Similar to the Variation of total fluorine concentration, the total lanthanum concentration is also maintained at a lower level at the pH values range from 4.3 to 8.2. When the pH value is over 12, the total lanthanum concentration increases, because lanthanum hydroxide is gradually converted into La (OH)_4_^-^ (Figure 5).

### 3.2 Fluorine removal from the sodium tungstate ion exchange effluent by chemical precipitation with lanthanum chloride

#### 3.2.1 Effect of lanthanum chloride amount on the fluorine removal

Experiments were carried out to examine the effect of lanthanum chloride amount on the removal ratio of fluorine with lanthanum chloride stoichiometric ratios ranging from 1.0 to 1.4 at a initial fluorine concentration of 105.6 mg/L, a pH value of 8.0, a reaction time of 30 min at 60°C.

**FIGURE 6:**
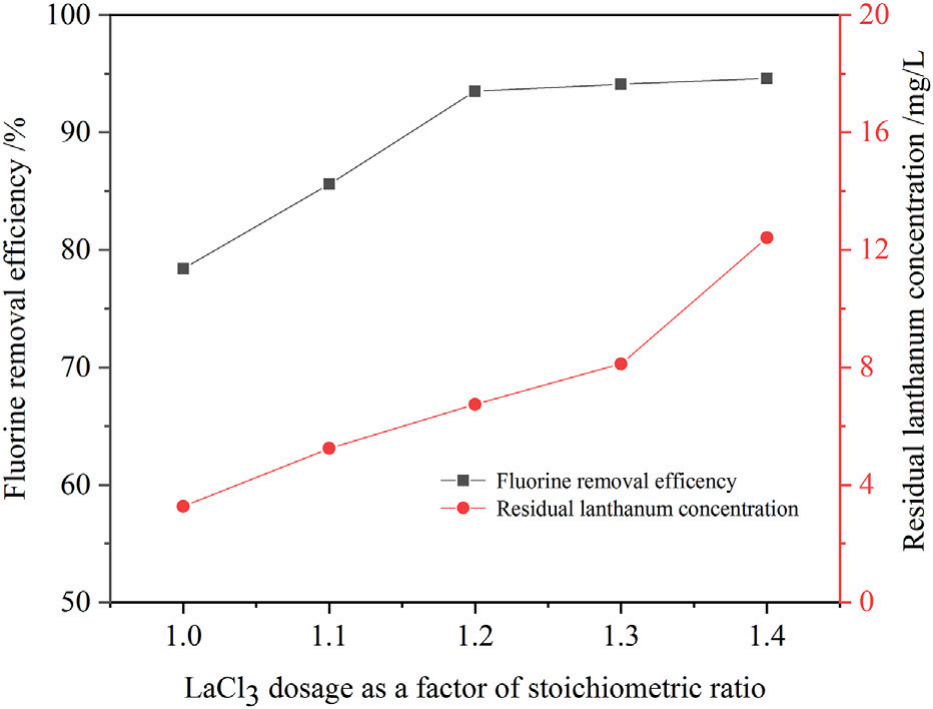
Effect of lanthanum chloride amount on the removal ratio of fluorine (initial fluorine concentration 105.6 mg/L, pH value 8.0, 30 min, 60°C).

Figure 6 shows that the dose of lanthanum chloride is an important factor for fluorine removal. When the dosage of lanthanum chloride increased from 1.0 times to 1.3 times the theoretical amount, the fluorine removal ratio increased from 78.4% to 94.1%, and the residual fluorine concentration in the solution decreased from 22.8 mg/L to 6.2 mg/L (Table 3). While, lanthanum concentration in the solution increase from 3.3 mg/L to 8.1 mg/L. When the lanthanum chloride dosage further incrased to 1.4 times the theoretical amount, the fluorine removal ratio almost unchanged, while the residual lanthanum concentration slightly incrased to 12.4 mg/L. When the dosage of lanthanum chloride is 1.3 times of the theoretical amount, the fluorine concentration in the solution after fluorine removal could meet the standard of national wastewater discharge [(F) = 10 mg/L]. Therefore, the suitable lanthanum chloride amount was determined to be 1.3 times the theoretical amount. Compared with the process of fluorine removal with calcium hydroxide, the efficiency of fluorine removal with lanthanum chloride was higher. Deep removal of fluorine in the sodium tungstate ion exchange effluent can be achieved through one-step precipitation operation. Hence, the new process has the advantages of high fluorine removal efficiency and simple operation.

**TABLE 3:** Main compositions of the ion exchange effluent after fluorine removal.

Elements	Na	Cl	F	P	As	Si	La
Concentration/mg/L	4,125	6,112	6.2	3.9	5.2	4.7	8.1

#### 3.2.2 Effect of solution pH value on the fluorine removal

**FIGURE 7:**
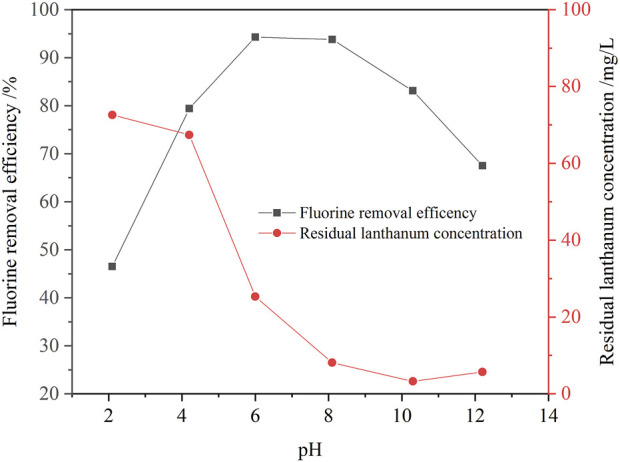
Effect of solution pH value on the removal ratio of fluorine (1.3 times the theoretical amount of lanthanum fluoride, initial fluorine concentration 105.6 mg/L, 30 min, 60°C).

In this study, the effect of solution pH value on the fluorine removal ratio was investigated, and results are shown in Figure 7. The solution pH value is also an important factor affecting the efficiency of fluorine removal. With the increase of solution pH value, the removal efficiency of fluorine first increased and then decreased. When the solution pH value increased from 2.1 to 8.0, the removal ratio of fluoride increased from 46.5% to 94.1%. While, when the pH value further increased to 12.2, the removal ratio of fluorine dramatically decreased to 67.5%. According to the above thermodynamic analysis of fluorine removal, when the solution pH value is lower than 2, lanthanum fluoride is not stable, and the fluorine concentration in the solution is higher. Therefore, when the solution pH value was 2.1, the removal ratio of fluoride removal was only 46.5%, and concentrations of residual fluorine and lanthanum in the solution were 56.5 mg/L and 72.6 mg/L, respectively. When the solution pH value was more than 10, lanthanum fluoride was gradually converted into lanthanum hydroxide, so the removal ratio of fluoride was reduced, and the concentration of fluorine ion was gradually increased. Therefore, the fluorine removal ratio was only 67.5% when the solution pH value was 12.2. When the solution pH value was 8.0, the lanthanum fluoride was stable in the solution, the removal ratio of fluorine reached 94.1%. In addition, the excess lanthanum ions were hydrolyzed into lanthanum hydroxide precipitates, so the residual lanthanium concentration in the solution was only 8.12 mg/L. Therefore, the optimal solution pH value was selected to be 8.0.

#### 3.2.3 Effect of temperature on the fluorine removal

**FIGURE 8:**
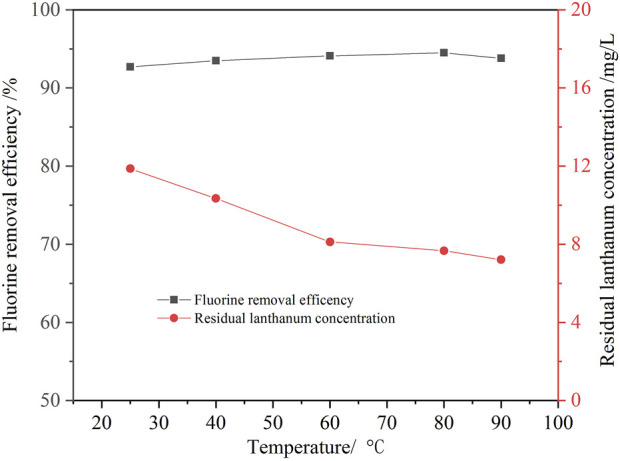
Effect of temperature on the removal ratio of fluorine (1.3 times the theoretical amount of lanthanum fluoride, initial fluorine concentration 105.6 mg/L, pH value 8.0, 30 min).

In this study, the effect of temperature on fluorine removal was also investigated. Figure 8 shows that the effect of temperature on the removal ratio of fluorine is slight. As the temperature increased from 10°C to 60°C, the fluorine removal ratio increased slightly from 91.6% to 94.1%, and the lanthanium concentration decreased from 11.9 mg/L to 8.1 mg/L. Increasing temperature was beneficial to the hydrolysis of lanthanum ions into lanthanum hydroxide, so the residual lanthanium concentration is lower. When the temperature was further increased to 90°C, the removal ratio of fluorine and residual lanthanium concentration changed little. Considering the removal ratio of fluorine and energy consumption, the optimal temperature was determined as 60°C.

#### 3.2.4 Effect of initial fluorine concentration on the fluorine removal

Since the fluorine concentration in the scheelite leaching solution (sodium tungstate) was variable, so the fluorine concentration in the ion exchange effluent also changed. Therefore, the effect of initial fluorine concentration on the fluorine removal was investigated.

**FIGURE 9:**
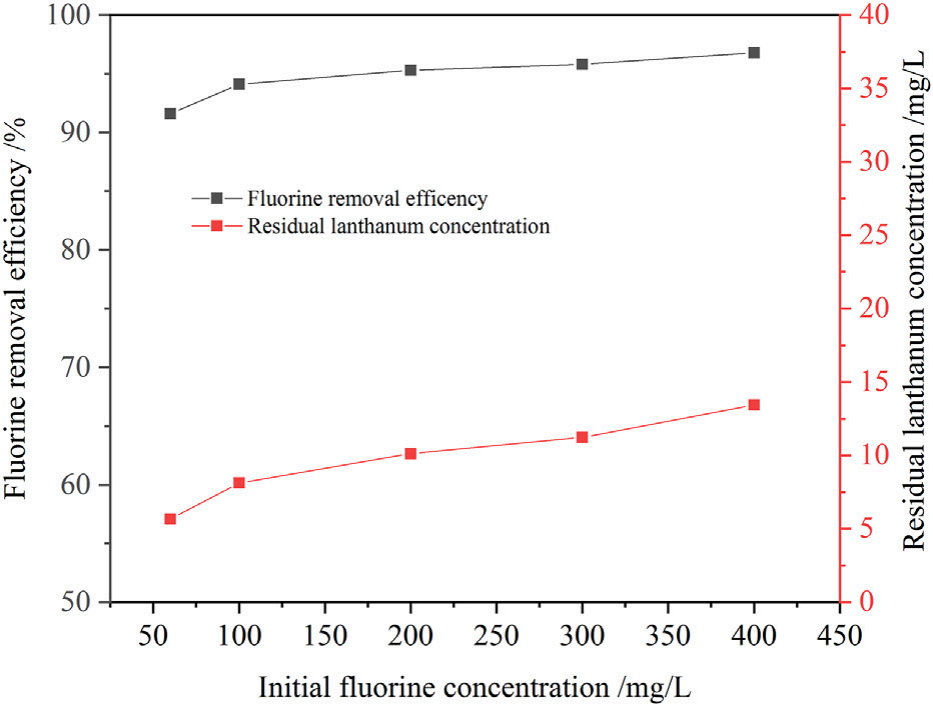
Effect of initial fluorine concentration on the removal ratio of fluorine (1.3 times the theoretical amount of lanthanum fluoride, pH value 8.0, 30 min, 60°C).

With the increase of initial fluorine concentration in the solution, the removal ratio of fluorine increased gradually (Figure 9). When the initial fluoride concentration increased from 60 mg/L to 400 mg/L, the fluorine removal ratio increased from 91.6% to 97.2%. Even if the initial fluorine concentration in the solution was as high as 400 mg/L, the fluorine concentration in the solution after fluorine removal was about 10 mg/L, which could meet the requirement of national wastewater discharge. It showed that the new process for remove fluorine from the ion exchange effluent with addition of lanthanum chloride had a good applicability. However, as the initial fluorine concentration in the solution increased, the residual lanthanum concentration also increased. This was because the amount of lanthanum chloride increased with the increase of the initial fluorine concentration, so the residual lanthanum concentration in the solution increased accordingly.

#### 3.2.5 Effect of reaction time on the fluorine removal

**FIGURE 10:**
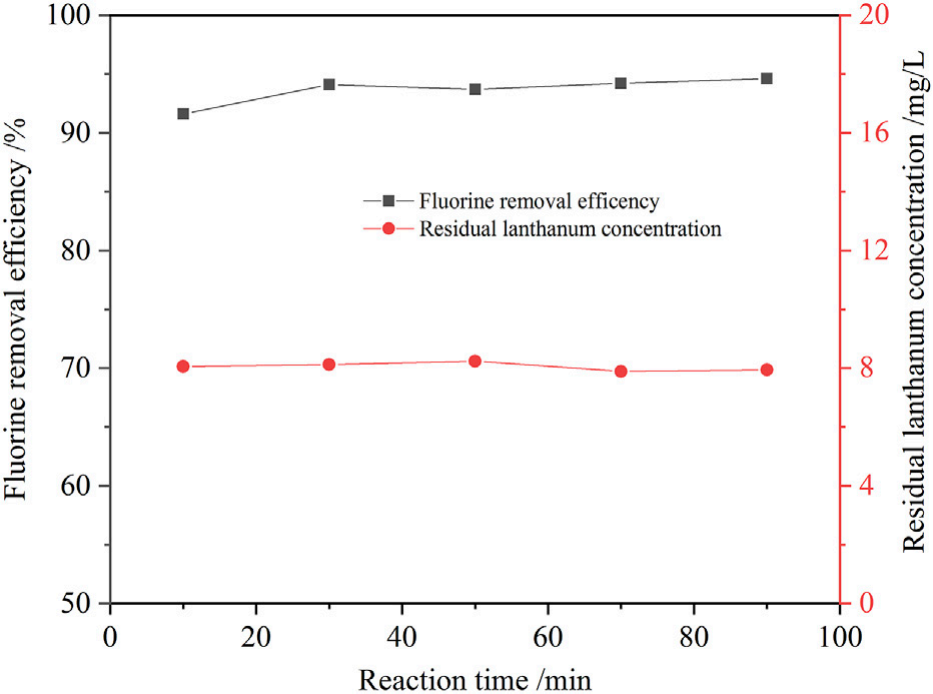
Effect of reaction time on the removal ratio of fluorine (1.3 times the theoretical amount of lanthanum fluoride, initial fluorine concentration 105.6 mg/L, pH value 8.0, 60°C).

**FIGURE 11:**
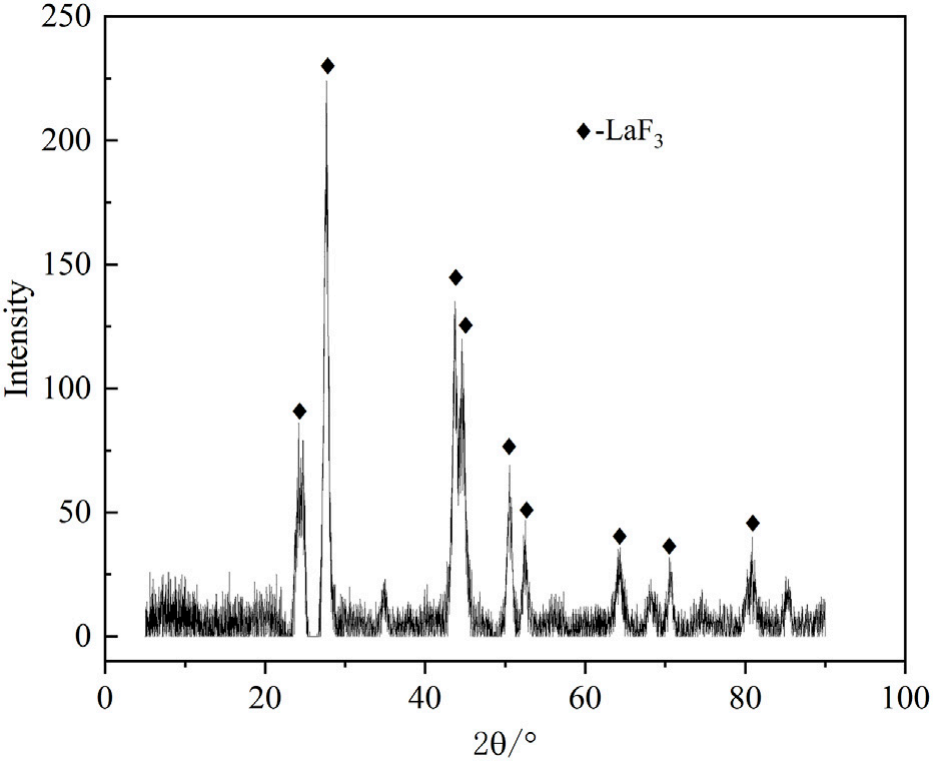
XRD patterns of the precipitated residue.

**FIGURE 12:**
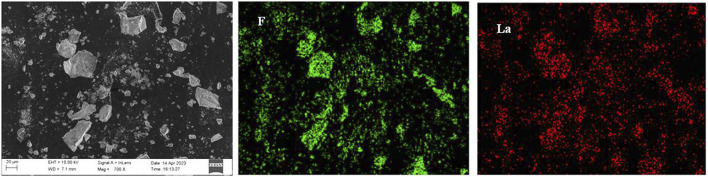
SEM and EDS of the precipitated residue.

The effect of the reaction time on the fluorine removal was also examined, and the results are shown in Figure 10. The reaction rate of fluoride removal from the ion exchange effluent was fast. Because the reaction of lanthanum chloride and fluoride ion to form lanthanum fluoride precipitates is a homogeneous reaction. Within 10 min, the fluorine removal ratio reached 91.6%. When the reaction time was extended to 30 min, the removal ratio of fluorine increased slowly to 94.1%. Further prolonging the reaction time, the removal ratio of fluorine was almost unchanged. The optimum reaction time to be 30 min upon taking the fluorine removal efficiency and manufacturing efficiency into full consideration. The defluorination residue was analyzed by XRD, SEM and EDS, and the results are shown in Figures 11, 12. Obvious diffraction peaks for lanthanum fluoride were found. The analysis results of the residue confirm that lanthanum fluoride was produced in the process of fluorine removal from the ion exchange effluent. The diffraction peaks of lanthanum hydroxide in the residue were not found, which may be the lanthanum hydroxide was amorphous.

## 4 Conclusion

In this study, an efficient method for removing fluorine from the sodium tungstate ion exchange effluent with addition of lanthanum chloride was developed. The thermodynamic analysis and process experiments of fluoride removal from the ion exchange effluent were carried out. The thermodynamic analysis results show that the reaction product lanthanum fluoride is stably formed at a pH value ranging from 1.0 to 10.0. The pH value of the solution beyonds this range is not conducive to fluorine removal. Experimental results showed that the dosage of lanthanum chloride and pH value of the solution had great influence on the fluorine removal. When the fluorine concentration in the solution was 60–400 mg/L, the removal ratio of fluorine was more than 92% under the optimal conditions: the lanthanum fluoride stoichiometric ratio of 1.3, the solution pH value of 8.0, temperature of 60°C and holding time of 30 min. After removing fluorine from the sodium tungstate ion exchange effluent, the residual fluorine concentration in the solution was lower than 10 mg/L, which could meet the standard of national wastewater discharge. The XRD, SEM and EDS analysis of residue indicated that reaction product was lanthanum fluoride with a low solubility product. Compared with traditional method of removing fluorine using calcium hydroxide, the new process has the obvious advantages of higher fluorine removal efficiency and lower reagent dosage.

## Data Availability

The original contributions presented in the study are included in the article/supplementary material, further inquiries can be directed to the corresponding author.
